# The diabetic interstitium as an overlooked layer of insulin resistance: vascular delivery, tissue transport, and insulin clearance

**DOI:** 10.3389/fendo.2026.1981356

**Published:** 2026-10-08

**Authors:** Renjie Wang, Xinxia Zhang

**Affiliations:** Chengdu University of Traditional Chinese Medicine, Chengdu, Sichuan, China

**Keywords:** endothelial transport, insulin clearance, insulin resistance, interstitium, microcirculation, skeletal muscle, type 2 diabetes

## Abstract

Insulin resistance is commonly framed as impaired receptor or post-receptor signaling, but that description begins only after insulin has reached a receptor-accessible compartment. *In vivo* insulin action also depends on delivery through the microcirculation, passage across the endothelium, distribution within interstitial fluid, and the balance between secretion and organ-specific clearance. This review integrates these upstream processes as an insulin-access continuum and asks whether diabetes can create a physiologically meaningful mismatch between plasma insulin and the insulin available to target cells. Human studies consistently show that skeletal-muscle interstitial insulin is lower than plasma insulin during hyperinsulinemia, although evidence that this gradient is uniformly greater in type 2 diabetes is mixed. Obesity, microvascular dysfunction, endothelial insulin resistance, extracellular-matrix remodeling, and altered tissue geometry can plausibly delay or spatially distort insulin access, but structural abnormalities alone do not prove a diffusion barrier. Clearance adds a second layer: reduced clearance may initially support compensation, whereas persistent hyperinsulinemia or, in selected phenotypes, excessive clearance may generate maladaptive exposure. We therefore view insulin resistance as a coupled exposure–response disorder rather than an exclusively intracellular defect. The framework is testable: informative studies should align plasma insulin and C-peptide kinetics with microvascular exchange, interstitial insulin, tissue glucose uptake, and downstream signaling. Such synchronized phenotyping can help distinguish causal access defects from compensation or association.

## 1 Introduction

Insulin resistance is classically defined as an inadequate biological response to a given insulin concentration. Most mechanistic work therefore focuses on the insulin receptor, IRS proteins, PI3K–AKT signaling, GLUT4 trafficking, mitochondrial metabolism, and other intracellular processes. Those mechanisms remain central, but they become relevant only after insulin has reached a receptor-accessible compartment. In the intact organism, insulin must first be presented to an exchange microvasculature, cross the endothelial interface, distribute through interstitial fluid and extracellular matrix, and remain available long enough to engage target cells. Vascular delivery and metabolic signaling are therefore linked parts of the same physiological process (1–3).

This upstream perspective is especially relevant in obesity and type 2 diabetes (T2D), where hyperinsulinemia can coexist with poor glucose disposal. Plasma insulin is easy to measure but does not directly report the concentration seen by a myocyte or adipocyte. Classic human microdialysis studies found that skeletal-muscle interstitial insulin remains well below plasma insulin during steady-state hyperinsulinemia (4). A matched study of people with T2D and healthy controls, however, found a similar plasma-to-interstitium gradient in both groups despite lower leg blood flow and glucose uptake in T2D (5). By contrast, obese women with postprandial hyperglycemia required higher arterial insulin concentrations to achieve interstitial insulin levels similar to lean controls, consistent with reduced fractional transfer into adipose tissue and muscle (6). Taken together, these studies argue against a universal ‘interstitial block’. Insulin access may be limiting in particular tissues, disease stages, or physiological states, whereas intracellular resistance may dominate in others.

Insulin clearance must be interpreted in the same integrated way. Hepatic first-pass extraction, renal removal, and peripheral uptake or degradation determine the magnitude and duration of systemic insulin exposure. Clearance varies with adiposity, hepatic metabolic state, disease stage, and insulin sensitivity, and it can change as dysglycemia develops (7–9). Lower clearance may temporarily preserve circulating insulin when sensitivity falls, whereas prolonged hyperinsulinemia can itself reshape vascular and metabolic signaling. Conversely, unusually high clearance can reduce insulin exposure despite substantial secretion (10). Secretion, access, clearance, and cellular responsiveness should therefore be measured as related variables rather than treated as interchangeable explanations.

Here we organize these processes into an insulin-access continuum spanning vascular presentation, endothelial transit, interstitial distribution, insulin removal, and receptor-level response. The main focus is obesity-associated insulin resistance and T2D. Skeletal muscle receives greatest attention because it is the best-studied peripheral tissue, while adipose tissue, liver, kidney, and cardiovascular tissues are considered according to their distinct anatomy and physiology. Human physiological evidence is prioritized where available; experimental studies are used to resolve mechanism. Throughout, we distinguish directly measured transport defects from structural or biochemical findings that only make such defects plausible. That distinction is essential because T2D extends well beyond glycemia, yet compartment-specific mechanistic claims still require compartment-specific evidence (11, 12).

## 2 The insulin-access continuum

### 2.1 An operational rather than anatomical model

We use the term insulin-access continuum for the sequence by which circulating insulin becomes an effective tissue signal. Regional flow and microvascular recruitment determine the opportunity for exchange; endothelial transit moves insulin from the vascular lumen to the abluminal space; interstitial transport governs its spatial and temporal distribution around target cells; clearance and degradation determine residence time; and receptor binding with post-receptor signaling converts exposure into metabolic action. This framework complements established cell-biological models rather than replacing them (3). **Figure 1** summarizes the sequence and the principal modifiers discussed below. Operational definitions for each component of the insulin-access continuum, together with preferred observables and common interpretive pitfalls, are summarized in **Table 1**.

**Figure 1:**
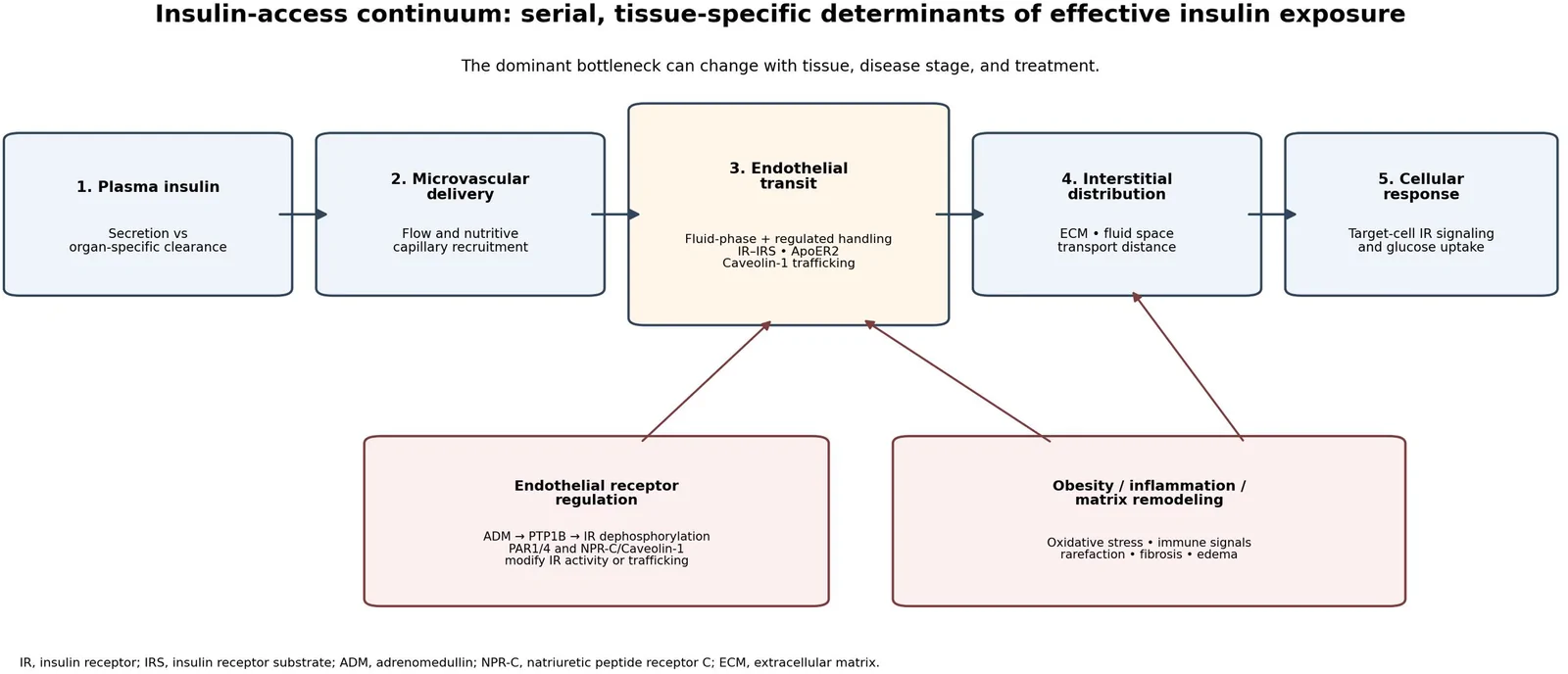
The insulin-access continuum. Circulating insulin must be delivered to exchange microvessels, cross the endothelium, distribute through interstitial space, and engage target-cell receptors. Endothelial IR/IRS signaling and associated trafficking machinery can regulate the transendothelial step, while obesity, inflammatory signals, matrix remodeling, and clearance modify exposure at several levels. The framework emphasizes serial, tissue-specific bottlenecks rather than a single universal barrier.

**Table 1:** Operational definitions within the insulin-access continuum.

Component	Operational definition	Preferred observable	Common interpretive pitfall
Vascular delivery	Presentation of insulin to nutritive exchange microvessels	Regional flow, capillary blood volume, microvascular recruitment, luminal insulin	Equating total limb flow with nutritive capillary exchange
Endothelial transit	Movement of insulin from capillary lumen to abluminal/interstitial space	Labeled insulin flux, endothelial uptake/transcytosis assays, paired vascular-interstitial kinetics	Assuming that more perfused capillaries guarantee rapid molecular transfer
Interstitial distribution	Movement and spatial distribution after endothelial exit	Calibrated microdialysis, lymph sampling, open-flow microperfusion, spatial transport measures	Treating matrix thickening or edema as direct proof of impaired insulin diffusion
Clearance	Irreversible removal from a defined compartment by hepatic, renal, or peripheral processes	C-peptide/insulin kinetic modeling, hepatic extraction estimates, organ-specific balance	Using a single fasting insulin:C-peptide ratio as a universal measure of clearance
Cellular responsiveness	Metabolic response to receptor-accessible insulin	Tissue glucose uptake, signaling phosphorylation, GLUT4 translocation	Attributing a whole-body insulin-sensitivity index to a single tissue or transport step

T2D, type 2 diabetes.

A single whole-body phenotype can arise from different bottlenecks along this sequence. During a hyperinsulinemic–euglycemic clamp, low glucose disposal might reflect poor capillary recruitment, delayed endothelial transit, altered interstitial distribution, excessive clearance, impaired post-receptor signaling, or a combination of smaller defects. Because the steps are serial, modest abnormalities at several levels can produce a larger kinetic delay than any one lesion alone. The continuum should therefore be used as a set of measurable hypotheses, not as evidence for a fixed anatomical barrier.

### 2.2 Concentration, time, and spatial heterogeneity

Plasma and tissue insulin are dynamically related. After a meal or infusion, plasma insulin changes rapidly, whereas tissue appearance is delayed by distribution and transport. At steady state, tissue exposure depends on the plasma driving concentration, transfer conductance, distribution volume, and removal; *in vivo*, all of these terms change with time. Studies showing that the route and temporal pattern of glucose or insulin administration alter measured insulin sensitivity illustrate why metabolic-test design matters (13, 14). Animal experiments likewise show that the timing of insulin exposure can influence later hepatic glucose disposal, reinforcing the point that insulin action depends on temporal context as well as concentration (15).

This timing creates an important clinical possibility: compensatory hyperinsulinemia can coexist with delayed or spatially heterogeneous tissue exposure. A high plasma concentration may partly overcome low transfer conductance, but it cannot show that every region of a tissue receives the same signal. Likewise, a normal or moderately elevated plasma insulin concentration can arise from high secretion with high clearance or from lower secretion with reduced clearance. Those states impose different beta-cell demands and may generate different tissue-exposure profiles even when a single blood sample appears similar.

## 3 Vascular delivery and endothelial transit

### 3.1 Microvascular recruitment is the first exchange gate

In skeletal muscle, insulin helps regulate its own delivery by recruiting nutritive microvascular units and expanding the endothelial surface available for exchange. This response is distinct from a large rise in total limb blood flow and may occur at lower insulin concentrations and earlier time points (2, 16). Microvascular responsiveness declines with aging and insulin resistance (17). In people with type 1 diabetes, skeletal-muscle microvascular recruitment has also correlated with insulin sensitivity, supporting the physiological relevance of this response without constituting direct evidence for T2D (18).

Effective exchange can be reduced by impaired endothelial nitric-oxide signaling, oxidative stress, inflammatory activation, capillary rarefaction, heterogeneous perfusion, and altered perivascular signaling. The concept of an ‘inflamed microvasculature’ links vascular injury to muscle insulin resistance (19, 20). High-fat feeding can impair coronary microvascular perfusion early in experimental metabolic disease (21), and perivascular adipose tissue can modify insulin-induced skeletal-muscle microvascular perfusion in humans (22). These observations place the microvasculature upstream of metabolic insulin action, while also underscoring that total blood flow and nutritive capillary exchange are not equivalent measurements.

### 3.2 Endothelial transit includes regulated IR-dependent components

Once insulin reaches perfused capillaries, passage across the endothelial layer becomes a distinct kinetic step. Current evidence does not support a single universal route. *In vivo* studies are compatible with fluid-phase exit from skeletal-muscle capillaries (23), whereas molecular and genetic studies show that endothelial signaling and trafficking machinery can regulate insulin uptake and transcytosis (24–30). Sun et al. used endothelial TFEB loss- and gain-of-function models to show that TFEB improves systemic glucose tolerance by increasing endothelial IRS1 and IRS2 and augmenting downstream AKT signaling; although transcytosis was not measured directly, the study establishes that the endothelial IR–IRS axis can influence whole-body insulin action (25). In obesity, Cho et al. showed that adrenomedullin activates PTP1B-dependent dephosphorylation of the endothelial insulin receptor. In cultured human microvascular endothelial cells, adrenomedullin reduced insulin uptake, insulin-induced receptor internalization, and insulin transcytosis; in obese mice, endothelial loss or blockade of the adrenomedullin receptor improved vascular insulin signaling, skeletal-muscle perfusion, and systemic insulin sensitivity (26). Rajala and Griffin provided complementary genetic evidence: endothelial Par1/4 deletion increased basal IR phosphorylation and insulin transcytosis, protected mice from streptozotocin-induced diabetes, and lost this protection when one endothelial Insr allele was additionally deleted (27). A recent review places these findings in the context of earlier endothelial Insr knockout studies and argues that IR activity is a regulatory determinant of insulin trafficking rather than a simple all-or-none gate (28).

Regulated endothelial handling is not limited to IR abundance itself. Endothelial ApoER2 deficiency reduces insulin uptake and skeletal-muscle delivery without altering direct insulin action in isolated muscle or insulin-stimulated microvascular recruitment, thereby separating transendothelial transport from myocyte responsiveness and capillary recruitment (29). More recently, endothelial NPR-C was shown to reduce IR membrane localization through Caveolin-1-dependent trafficking; endothelial NPR-C deletion improved insulin uptake, transendothelial transport, and systemic insulin sensitivity, whereas endothelial overexpression had the opposite effect (30). Taken together, fluid-phase and receptor-regulated processes may coexist, with their relative contributions depending on tissue, insulin concentration, endothelial phenotype, species, and disease state.

### 3.3 Direct evidence: what human and animal perturbation studies actually show

Direct access to the interstitium provides some of the strongest evidence that delivery can become rate limiting. In healthy humans, calibrated microdialysis showed muscle interstitial insulin concentrations approximately 30–50% below simultaneous plasma concentrations during hyperinsulinemic clamps (4). In anesthetized dogs, direct intramuscular insulin administration largely removed the delay between insulin exposure and hindlimb glucose uptake, supporting the capillary–endothelial step as an important determinant of the time course of insulin action (31). The human T2D study noted above, however, found no significant difference in steady-state interstitial insulin between T2D and matched controls despite lower glucose uptake in T2D (5). That result is an important counterweight to the idea that impaired tissue access universally explains peripheral insulin resistance. Representative human and experimental evidence relevant to impaired insulin access is summarized in **Table 2**.

**Table 2:** Representative evidence relevant to impaired insulin access.

Evidence line	Model/tissue	Main observation	Interpretation for the access hypothesis
Calibrated muscle microdialysis (4)	Healthy humans, skeletal muscle	Interstitial insulin remained markedly below plasma insulin during clamp hyperinsulinemia	Supports a physiologic plasma-to-interstitium gradient and a transport delay
Matched T2D-control microdialysis (5)	Humans with T2D vs controls	Interstitial insulin did not differ significantly between groups despite lower glucose uptake in T2D	Argues against a universal transcapillary defect as the dominant cause of T2D muscle insulin resistance
Postprandial tissue microdialysis (6)	Obese women with postprandial hyperglycemia	Higher arterial insulin was required to achieve interstitial levels similar to lean controls	Supports impaired fractional insulin transfer in an obesity-associated phenotype
Direct intramuscular insulin (31)	Dogs, skeletal muscle	Bypassing vascular delivery markedly shortened the delay in glucose uptake	Strong experimental support that delivery can limit the time course of insulin action
Endothelial ApoER2 manipulation (29)	Mice and endothelial cells	Reduced endothelial transport lowered muscle insulin delivery and glucose disposal without changing direct myocyte insulin action	Separates endothelial transport from intrinsic muscle responsiveness
Fluid-phase capillary exit (23)	Experimental skeletal-muscle microvasculature	Insulin capillary exit was consistent with fluid-phase transport	Challenges a universally receptor-saturable transcytosis model
Endothelial IR/IRS genetic regulation (25–28)	Mice and human endothelial cells	TFEB–IRS1/2, adrenomedullin–PTP1B–IR, and PAR1/4 perturbations altered endothelial insulin signaling; PAR1/4 loss increased insulin transcytosis in an Insr-dependent manner	Supports a regulated IR-dependent component of endothelial insulin handling without excluding fluid-phase transport or perfusion effects
Endothelial NPR-C/Caveolin-1 manipulation (30)	Obese mice and endothelial cells	NPR-C altered Caveolin-1 stability and IR membrane localization; endothelial NPR-C deletion improved insulin uptake, transendothelial transport, and insulin sensitivity	Links receptor membrane trafficking directly to endothelial insulin transport in obesity

Evidence strength differs by species, tissue, and whether insulin flux was measured directly.

Perturbation studies provide mechanistic leverage but do not always prove mediation. GLP-1 and insulin regulate skeletal and cardiac muscle microvascular perfusion in T2D (32). The pan-AMPK activator O304 improved glucose homeostasis together with microvascular perfusion in experimental and clinical settings (33), while salsalate attenuated free-fatty-acid-induced microvascular and metabolic insulin resistance in humans (34). Globular adiponectin improves microvascular insulin responses through AMPK-dependent mechanisms in experimental models (35). These parallel vascular and metabolic effects are compatible with an access mechanism, but formal mediation requires evidence that an intervention changes insulin arrival in tissue and that this change quantitatively explains improved glucose disposal.

Endothelial biology is also shaped by immune and metabolic signals. Hyposialylated IgG can activate endothelial FcγRIIB and promote obesity-associated insulin resistance (36), while a branched-chain amino-acid metabolite can drive vascular fatty-acid transport and insulin resistance (37). The endothelium is therefore more than a passive conduit: it is a signaling interface capable of modifying hormone and substrate exchange.

## 4 The interstitial compartment: barrier, buffer, or bystander?

### 4.1 Extracellular matrix and tissue geometry

After crossing the endothelium, insulin enters a hydrated and structurally heterogeneous compartment containing basement membrane, extracellular matrix (ECM), interstitial fluid, stromal cells, immune cells, nerves, and tissue-specific diffusion distances. The relevant question is not simply whether insulin is detectable outside the vessel, but whether receptor-accessible concentrations rise at the right place and time. Diabetes and obesity remodel vascular ECM and basement membrane, changes increasingly recognized as active features of microvascular disease rather than inert histological consequences (38). Greater diffusion distance, altered pore structure, nonenzymatic glycation, fibrosis, edema, and heterogeneous capillary spacing could therefore modify insulin distribution.

Structural plausibility, however, is not proof of a transport barrier. Basement-membrane thickening can accompany impaired exchange without being the rate-limiting cause; fibrosis can change tissue mechanics or perfusion without directly restricting insulin diffusion; and increased nonspecific permeability can increase leakage while worsening spatial distribution. A strong causal test would measure matrix or fluid structure, insulin transport, and tissue metabolism in the same tissue, then show that correcting the structural abnormality changes insulin exposure before glucose uptake improves. Few studies currently meet all three requirements.

### 4.2 Inflammation, fluid balance, and interstitial chemistry

Inflammation can couple vascular and interstitial dysfunction through endothelial activation, altered permeability, immune-cell recruitment, matrix deposition, and changes in tissue fluid balance. It can also affect insulin passage more directly by changing receptor function. In obesity, circulating adrenomedullin is increased and activates a Gs/PKA/PTP1B pathway that dephosphorylates endothelial IR; Cho et al. found that this pathway reduced insulin uptake, receptor internalization, and transcytosis in human microvascular endothelial cells, while endothelial loss or blockade of the adrenomedullin receptor improved insulin signaling and systemic sensitivity in obese mice (26). Experimental diabetes provides a second genetic example: endothelial PAR1/4 deletion increased IR phosphorylation and insulin transcytosis in an Insr-dependent manner (27). These receptor-level effects can coexist with changes in permeability, perfusion, and matrix structure. Adipose tissue is especially relevant because immune–stromal crosstalk, adipocyte hypertrophy, fibrosis, and altered lipid flux can affect both local and systemic insulin action. Work focused on type 1 diabetes also supports the broader principle that adipose immune cells and tissue structure modify metabolic signaling rather than acting as isolated compartments (39).

Interstitial chemistry may add another layer, although the evidence remains much less mature. Low interstitial-fluid pH has been reported in diabetic animal tissue (40), and mechanistic hypotheses have linked interstitial acidification to glycolytic and mitochondrial dysfunction (41, 42). These findings are interesting, but they do not yet establish interstitial pH as a clinically important regulator of insulin transport in human skeletal muscle or adipose tissue. It should therefore be presented as an emerging hypothesis rather than a validated therapeutic target.

### 4.3 Tissue specificity prevents a single ‘diabetic interstitium’

The anatomy of insulin access differs substantially across organs. Skeletal muscle has continuous capillary endothelium and is the clearest setting in which microvascular exchange can constrain insulin-stimulated glucose uptake. Adipose tissue combines microvascular control with large changes in cell size, ECM, inflammation, and lipolysis. The liver is fundamentally different because sinusoidal exchange and first-pass insulin extraction are integral to its physiology, while the kidney is both insulin responsive and an important site of insulin removal. ‘Diabetic interstitium’ is therefore best used as a unifying physiological concept, not as a claim that every tissue shares one anatomical lesion.

Imaging of perivascular-space fluid dynamics in prediabetes and T2D illustrates the growing ability to interrogate interstitial compartments *in vivo* (43), but neuroimaging-derived fluid measures cannot be extrapolated directly to skeletal-muscle insulin diffusion. A method can establish that a compartment is altered without showing that the alteration limits insulin transport.

## 5 Insulin clearance and degradation: compensation, cause, and consequence

### 5.1 Clearance is an exposure regulator

Systemic insulin concentration reflects secretion minus removal. Hepatic first-pass extraction is the dominant early determinant, with renal and peripheral uptake or degradation contributing according to physiological state. In obesity and T2D, clearance is closely linked to insulin sensitivity, beta-cell secretion, hepatic lipid content, and nutritional state (7, 44). Insulin-degrading enzyme (IDE) contributes to hepatic insulin and glucose homeostasis in diet-induced obesity (45), while prolonged high-fat feeding can reduce renal IDE expression and activity through PPARγ-related mechanisms (46). IDE abundance in liver or kidney is not, however, synonymous with whole-body clearance, which also depends on secretion, receptor-mediated uptake, blood flow, organ extraction, and feedback.

### 5.2 Why reduced clearance can be adaptive

When insulin sensitivity begins to fall, lower clearance can help sustain circulating insulin and reduce the additional secretory demand needed to preserve glucose homeostasis. Longitudinal cohorts show that clearance changes over time rather than behaving as a fixed personal trait (8, 47), and studies of early dysglycemia show that its contribution is interpretable only when secretion and sensitivity are estimated together (9). Even mild experimental hyperglycemia can shift the balance among secretion, clearance, and sensitivity, illustrating how quickly these variables respond to the metabolic environment (48).

Lower clearance may therefore be useful early in insulin resistance because it prolongs circulating insulin and reduces the immediate burden on beta cells. The same response may become less helpful if hyperinsulinemia persists. Tissues that remain partly insulin responsive are then exposed to more hormone for longer, potentially affecting hepatic lipid handling, vascular signaling, and beta-cell workload. Human observations are consistent with this stage-dependent interpretation: in recent-onset diabetes, the relation between clearance, liver fat, and glycemic control differs across metabolic phenotypes (49), and lower clearance has been associated with subclinical atherosclerosis in people at risk for T2D (50). These associations do not show that low clearance directly causes vascular disease because adiposity, liver dysfunction, and insulin resistance influence the same measurements.

### 5.3 Increased clearance and stage-specific heterogeneity

The opposite phenotype—relatively high clearance—can also reduce peripheral insulin exposure. Increased hepatic insulin clearance has been reported in selected patients with poorly controlled T2D (51), and a case report shows that unusually high clearance can coexist with preserved secretion and insulin sensitivity (10). Diabetes should therefore not be described as a uniformly low-clearance state. The Swedish CArdioPulmonary BioImage Study (SCAPIS) illustrates the value of integrated metabolic–vascular phenotyping. In a cross-sectional analysis of 2,054 adults aged 50–64 years without diabetes from the Umeå SCAPIS site, OGTT-derived indices of insulin sensitivity, secretion, and clearance were examined alongside coronary computed tomography angiography and carotid ultrasound (52). Lower insulin sensitivity remained associated with higher coronary artery calcium and segment involvement scores after adjustment, whereas associations between lower clearance and coronary atherosclerosis were attenuated after adjustment for cardiovascular risk factors. This pattern shows why sensitivity, secretion, clearance, and vascular phenotype should be analyzed together, while also emphasizing that cross-sectional clearance indices cannot establish causal direction.

Intervention studies further illustrate this context dependence. Short-term SGLT2 inhibition did not change systemic insulin clearance in one study (53), whereas ipragliflozin improved liver function and insulin resistance in Japanese patients with T2D (54), and tofogliflozin-associated increases in hepatic clearance tracked changes in triglycerides and beta-hydroxybutyrate (55). Bariatric procedures alter fasting and prandial insulin clearance (56). Differences among studies may reflect treatment duration, weight loss, liver fat, meal state, glycemic control, and the method used to infer clearance. The context-dependent insulin-clearance phenotypes, their potential consequences, and the measurements required for interpretation are summarized in **Table 3**.

**Table 3:** Interpreting insulin-clearance phenotypes.

Observed phenotype	Possible short-term meaning	Potential long-term consequence	What must be measured concurrently
Reduced clearance with preserved secretion	Compensatory increase in systemic insulin exposure	Persistent hyperinsulinemia; tissue-selective overexposure; uncertain vascular effects	C-peptide-derived secretion, hepatic fat, insulin sensitivity, tissue exposure
Reduced clearance with hepatic metabolic dysfunction	Consequence of altered liver handling rather than primary adaptation	Further uncoupling of portal and peripheral insulin exposure	Hepatic extraction, liver fat/function, portal-proxy kinetics
Increased clearance with robust secretion	Lower peripheral exposure despite beta-cell compensation	Higher secretory demand and relative tissue insulin deficiency in susceptible tissues	Secretion rate, systemic clearance, tissue insulin, glucose disposal
Treatment-associated clearance change	Part of a multicomponent metabolic response	May be beneficial, neutral, or compensatory depending on sensitivity and secretion	Before/after secretion, clearance, body weight, hepatic metabolism, meal state

Clearance should be interpreted as a dynamic boundary condition rather than labeled uniformly as beneficial or harmful.

## 6 Coupled failure across tissues and disease stages

### 6.1 Skeletal muscle and adipose tissue

Skeletal muscle provides the clearest conceptual link between insulin access and glucose disposal because insulin must cross a continuous microvascular endothelium before reaching myocytes. Delivery is only part of the problem, however. Rac1 deficiency, for example, worsens high-fat-diet-induced impairment of insulin-stimulated muscle glucose uptake independently of Akt, showing that intracellular defects can remain limiting even when hormone delivery is adequate (57). An access model should therefore complement, not minimize, intrinsic myocyte insulin resistance.

Adipose tissue adds another set of feedbacks. Expansion can alter capillary density, perfusion, matrix composition, inflammatory-cell populations, and lipolysis. The resulting free-fatty-acid and cytokine flux can then worsen muscle and hepatic insulin resistance. Human studies show that tissue-specific contributors to obesity-associated insulin resistance can be separated only imperfectly, which strengthens the case for multi-organ phenotyping (58). Adipose tissue is both a target of access defects and a source of signals capable of creating vascular and cellular resistance elsewhere.

### 6.2 Liver, kidney, and cardiovascular tissues

The liver sits at the intersection of insulin exposure and insulin removal. It receives portal insulin, performs first-pass extraction, regulates endogenous glucose production, and links hepatic lipid accumulation with systemic metabolic disease. Genetic and molecular work on fatty liver and diabetes emphasizes the bidirectional relationship between hepatic metabolism and dysglycemia (59), while human clamp studies demonstrate that hepatic glucose uptake during hyperinsulinemia relates to glycemic phenotype (60). Because portal and peripheral insulin concentrations differ, a peripheral blood sample is especially poor at localizing a hepatic defect.

The kidney contributes to insulin removal and is simultaneously vulnerable to diabetes-related microvascular disease. Cardiovascular tissues provide another link between vascular insulin resistance and clinical outcomes, but associations between clearance, sensitivity, and subclinical atherosclerosis require cautious causal interpretation (50, 52). These organ-specific roles explain why a single value such as fasting insulin cannot distinguish high secretion with high removal from moderate secretion with low removal, nor identify tissue-selective access defects.

### 6.3 A disease-stage model

A stage-based model helps reconcile findings that otherwise seem contradictory. Early in metabolic dysfunction, beta-cell compensation and reduced clearance may maintain glycemia despite emerging vascular or cellular resistance. With progression of obesity and T2D, endothelial dysfunction, inflammation, matrix remodeling, hepatic lipid accumulation, and changing clearance can widen the mismatch between plasma insulin and effective tissue exposure. Later beta-cell failure lowers the driving insulin concentration and may make pre-existing access limitations more consequential. This sequence is physiologically plausible, but no longitudinal human study has yet measured every component of the continuum on the same time axis.

The framework also predicts treatment heterogeneity. A person whose dominant limitation is microvascular delivery should not be assumed to respond in the same way as someone with high hepatic clearance or severe post-receptor resistance. This is a rationale for mechanism-based phenotyping, not a current clinical classification. Any proposed phenotype should first show reproducibility, an independent relation to tissue insulin action, and differential response to a targeted perturbation.

## 7 Measuring the insulin-access continuum

### 7.1 Integrated metabolic tests are powerful but poorly localizing

The hyperinsulinemic-euglycemic clamp remains a robust measure of insulin-mediated glucose disposal, but it integrates rather than localizes the physiology. An abnormal glucose infusion rate cannot identify whether the dominant defect lies in delivery, transport, clearance, or intracellular response. Minimal-model approaches similarly infer insulin sensitivity and glucose effectiveness from dynamic concentration data but necessarily rely on compartmental assumptions (61). Surrogate indices can also be influenced by hepatic insulin clearance, which is one reason indices designed to reduce clearance-related bias have been explored (62).

Protocol details matter. Insulin dose, route of glucose administration, meal state, time of day, and sampling schedule can alter the relative contribution of different physiological processes (13, 63). The goal is not to replace conventional insulin-sensitivity measures, but to decompose them with adjunct measures when a specific access mechanism is being tested.

### 7.2 Direct and near-direct measurement of vascular and interstitial processes

Microvascular perfusion can be assessed with contrast-enhanced ultrasound and related imaging approaches. Multiscale and morphological analysis of contrast-enhanced ultrasound, including super-resolution methods, can improve description of microvascular networks (64, 65). These tools estimate exchange opportunity, not insulin flux itself. Calibrated microdialysis, open-flow microperfusion, lymph sampling, labeled insulin kinetics, and paired arterial-interstitial sampling provide more direct access to tissue insulin exposure, but each has limitations in recovery, calibration, perturbation, spatial coverage, and temporal resolution.

No available technique should be described as a direct measure of receptor-site insulin concentration unless that relationship has been validated. Microdialysis samples a small region and recovery depends on peptide properties; lymph integrates a larger drainage territory but has slow kinetics; biopsy provides discrete time points and mixes vascular, interstitial, and cellular pools; and imaging generally measures perfusion rather than molecular transport. The most informative studies will combine complementary methods rather than treating one readout as definitive.

### 7.3 A minimum dataset for causal inference

For a study to claim that impaired insulin access causes insulin resistance, a practical minimum dataset should include plasma insulin and C-peptide kinetics, a validated estimate of systemic clearance, a measure of microvascular exchange, a direct or near-direct estimate of interstitial insulin, and a tissue-specific metabolic endpoint. A measurement matrix linking key mechanistic questions to candidate methods, strengths, and major limitations is provided in **Table 4**. Where feasible, tissue morphology and post-receptor signaling should be added to distinguish access from response. Standard reporting should also specify disease stage, adiposity, medications, fasting or meal state, insulin dose and route, tissue sampled, timing, and the assumptions used to estimate secretion and clearance.

**Table 4:** Measurement matrix for mechanism-based studies.

Question	Candidate methods	Strength	Major limitation
How much insulin is presented to exchange microvessels?	Regional flow, contrast-enhanced ultrasound, capillary blood volume	Spatially resolves exchange opportunity	Does not directly measure insulin molecules crossing the endothelium
How fast does insulin cross endothelium?	Labeled insulin kinetics, endothelial transport assays, arterial-interstitial time curves	Targets the endothelial step	Species/model dependence; difficult in human tissue
What insulin concentration exists in interstitial fluid?	Calibrated microdialysis, open-flow microperfusion, lymph sampling	Near-direct tissue-exposure estimate	Sampling recovery and limited tissue volume or slow lymph kinetics
How much insulin is secreted versus cleared?	Insulin plus C-peptide deconvolution, organ extraction modeling	Separates secretion from systemic concentration	Model assumptions and difficulty resolving organ-specific clearance
Does access affect metabolism?	Clamp plus tissue glucose uptake and signaling measures	Links exposure to biological effect	Requires synchronized multimodal sampling

Method selection should be driven by the compartmental claim being tested.

## 8 Therapeutic implications: use the framework to choose endpoints, not treatments

### 8.1 Exercise and vascular-metabolic interventions

Exercise acts across several levels at once, improving endothelial function, perfusion, mitochondrial metabolism, adiposity, and intracellular insulin signaling. Physical activity has been associated with skeletal-muscle insulin sensitivity after bariatric surgery (66), but that association does not identify which access component changed. Studies designed to test an access mechanism should therefore pair exercise with microvascular and interstitial measurements.

Vascular-active interventions provide more focused perturbations. High-dose tadalafil has been tested in well-controlled T2D as a pharmacological probe of vascular contributions to insulin resistance (67). GLP-1-related microvascular regulation (32), O304-mediated AMPK activation (33), and anti-inflammatory salsalate (34) similarly link vascular and metabolic endpoints. Metformin plus sulodexide restored cardiac microvascular perfusion capacity in diet-induced obese rats (68), although a cardiac perfusion effect in rodents should not be generalized directly to skeletal-muscle insulin delivery in humans.

### 8.2 Metabolic drugs, diet, and bariatric surgery

Most metabolic treatments affect several nodes of the continuum. Imeglimin, for example, influences both insulin secretion and sensitivity (69). Sodium–glucose cotransporter 2 (SGLT2) inhibitors block SGLT2 in the renal proximal tubule, thereby reducing glucose reabsorption and increasing urinary glucose excretion. Because their primary glucose-lowering effect does not require insulin, subsequent changes in insulin demand, hepatic metabolism, body weight, substrate use, or insulin clearance are secondary and may differ by setting (53–55). Tauroursodeoxycholic acid (TUDCA) is a hydrophilic bile-acid conjugate that acts as a chemical chaperone in experimental systems, helping reduce endoplasmic-reticulum stress and stabilize protein-folding homeostasis. In mice, TUDCA has also reduced age-related hyperinsulinemia (70), but its pleiotropic effects do not establish a clinically validated insulin-clearance therapy for T2D. Mechanistic placement therefore requires direct measurement of the proposed node rather than inference from a fall in glucose or insulin alone.

Diet and meal timing can alter secretion, clearance, and insulin demand at the same time. Low-carbohydrate and Mediterranean dietary patterns have been compared for effects on glucose metabolism, insulin kinetics, and beta-cell function in severe obesity (71), and early time-restricted carbohydrate consumption has been tested in T2D (72). Bariatric surgery changes insulin clearance in both fasting and prandial states (56). Improvements in glycemia or a clamp-derived index therefore cannot, by themselves, demonstrate correction of a vascular or interstitial barrier.

### 8.3 Matrix, inflammation, and integrative interventions

Inflammation- or matrix-directed strategies could theoretically improve endothelial exchange and tissue distribution, but mechanistic claims should depend on whether the proposed layer was actually measured. The same standard applies to integrative interventions. Statements that a therapy ‘improves microcirculation’, ‘resolves stasis’, or normalizes the tissue environment should be translated into prespecified physiological endpoints—such as capillary recruitment, endothelial transport, interstitial insulin kinetics, matrix composition, or clearance—rather than inferred from glucose lowering alone.

A multicomponent trial can still be mechanistically rigorous if it combines a conventional metabolic endpoint with perfusion imaging, insulin/C-peptide kinetic modeling, and a calibrated interstitial measure. The aim is not to force a complex intervention into one pathway, but to determine which physiological layers changed and whether they changed in the temporal sequence predicted by the access model. Representative intervention classes, their potential access-related actions, and the endpoints required for stronger causal attribution are summarized in **Table 5**.

**Table 5:** Mapping interventions onto the insulin-access continuum.

Intervention class	Potential access-related action	What current evidence can support	Endpoint needed for stronger causal attribution
Exercise/physical activity (66)	Capillary recruitment, endothelial function, mitochondrial and cellular insulin action	Improved insulin sensitivity with broad vascular-metabolic effects	Simultaneous perfusion, interstitial insulin, and muscle glucose uptake
PDE5/vascular-active therapy (67)	Vasodilation and microvascular exchange	Feasibility and metabolic effects in T2D	Direct insulin-delivery or interstitial-exposure measurement
GLP-1/AMPK-active approaches (32, 33)	Microvascular recruitment plus metabolic signaling	Parallel perfusion and glucose-homeostasis improvement	Mediation by tissue insulin arrival
Anti-inflammatory or glycocalyx/matrix-oriented approaches (34, 68)	Reduced endothelial inflammation; improved microvascular function	Vascular and metabolic perturbation in human or experimental settings	Structure/transport/metabolism linkage in the same tissue
SGLT2 inhibitors and bariatric surgery (53, 55, 56)	Indirect effects through glycemia, liver metabolism, weight, and clearance	Context-dependent changes in insulin kinetics	Secretion-clearance decomposition and tissue exposure
Diet/meal timing (71, 72)	Changes in insulin demand, secretion, substrate flux, and possibly clearance	Altered glucose metabolism and insulin kinetics	Time-resolved secretion, clearance, and tissue-access measures

Therapeutic plausibility should not be interpreted as a treatment recommendation.

## 9 Discussion

### 9.1 What the evidence supports—and what it does not

The available evidence supports a broader view of insulin resistance in which effective insulin action depends on both hormone exposure and cellular response. The vascular component is well established physiologically, particularly in skeletal muscle, and recent genetic work shows that endothelial IR signaling and trafficking machinery can alter insulin delivery without changing direct myocyte responsiveness (25–30). Human measurements confirm a substantial plasma-to-interstitium insulin gradient. Clearance is also an established determinant of systemic exposure and varies dynamically across obesity and T2D. What remains uncertain is whether a structurally defined ‘diabetic interstitial barrier’ is a common, independent cause of T2D insulin resistance.

Direct human evidence remains mixed. A lower interstitial than plasma insulin concentration is a normal physiological feature, and at least one matched T2D study did not find a further reduction in steady-state interstitial insulin (5). In contrast, obesity-associated postprandial hyperglycemia has been linked to a lower fraction of circulating insulin reaching adipose and muscle interstitial fluid (6). These observations can coexist if transport defects are stage-specific, stimulus-specific, or masked at steady state by higher plasma insulin. Kinetic delay and reduced fractional transfer may therefore be more informative than a single steady-state concentration.

### 9.2 Principal controversies

Three controversies should remain explicit. First, endothelial insulin transit can include both fluid-phase and regulated, IR-dependent components, and their relative contribution is likely to vary by tissue and disease context (23–30). Second, ECM and basement-membrane remodeling provide a plausible physical basis for altered distribution but have not been linked consistently to quantitative insulin flux in humans (38). Third, reduced insulin clearance may be adaptive, maladaptive, or sequentially both; cross-sectional associations cannot establish direction (7, 50, 52). Rather than resolving these issues rhetorically, a useful review should state the competing interpretations and identify experiments capable of distinguishing them. The principal controversies, competing explanations, and experiments capable of distinguishing among them are summarized in **Table 6**.

**Table 6:** Controversies and decisive experiments.

Controversy	Competing explanations	Decisive experiment
Does endothelial transit materially limit insulin action in T2D?	Context-dependent mixture of fluid-phase and regulated IR-dependent transport vs dominant myocyte resistance	Endothelial IR-pathway perturbation combined with high-temporal-resolution plasma/interstitial insulin, microvascular exchange, muscle glucose uptake, and signaling
Does ECM remodeling create an insulin diffusion barrier?	Matrix directly restricts insulin vs matrix is a marker of broader tissue disease	Intervention that reverses a quantified matrix feature and demonstrates earlier/faster insulin arrival before improved metabolism
Is reduced clearance protective or pathogenic?	Compensation for low sensitivity vs driver of chronic hyperinsulinemia	Longitudinal/perturbation study separating secretion, hepatic extraction, peripheral exposure, and vascular/metabolic outcomes
Are microvascular improvements causal for glucose disposal?	Mediation by insulin delivery vs parallel vascular and metabolic effects	Intervention with formal temporal mediation using tissue insulin arrival as intermediate endpoint
Can mechanism-based phenotypes guide therapy?	Stable biological subtypes vs transient states driven by disease stage and treatment	Prospective reproducibility study followed by treatment-by-phenotype interaction testing

These experiments are designed to falsify, not merely illustrate, the insulin-access model.

### 9.3 A practical research agenda

The highest priority is a synchronized human study that measures the full continuum on a common time axis. Plasma insulin and C-peptide, systemic and preferably hepatic clearance, capillary exchange, interstitial insulin, tissue glucose uptake, and post-receptor signaling should be sampled densely enough to resolve delays. Steady-state values alone can miss a primarily kinetic transport defect. Longitudinal designs are then needed to determine whether an access phenotype precedes worsening glucose tolerance, emerges as compensation, or appears only after established T2D.

A second priority is tissue-specific validation. Methods established in skeletal muscle cannot simply be transferred to liver or adipose tissue because endothelial architecture, insulin extraction, and physiological function differ. Kinetic models should distinguish redistribution from irreversible removal and report parameter identifiability. Intervention studies should then perturb one candidate layer while measuring the others, so that compensation and mediation can be tested rather than assumed.

Mechanism-based stratification also deserves prospective evaluation. Participants should be classified by dominant delivery, clearance, or post-receptor abnormalities only after the relevant measures prove reproducible. A useful phenotype must then predict differential response to a targeted intervention. Until those criteria are met, the continuum is better used to sharpen experimental design and interpretation than to label patients.

### 9.4 Limitations of the current evidence

The evidence base is heterogeneous across species, tissue, metabolic stage, adiposity, insulin dose, nutritional state, transport method, and clearance model. Many studies measure one segment of the continuum and infer what happens in adjacent segments. Direct human interstitial-insulin studies are small and technically demanding, while much of the molecular endothelial literature remains experimental. Structural observations in the interstitium often lack simultaneous insulin-flux measurements, and clearance studies are particularly vulnerable to reverse causation. These limitations prevent a reliable quantitative estimate of how much whole-body insulin resistance is attributable to impaired access.

This review is mechanistic rather than systematic. It integrates human physiological studies with animal and cellular experiments to define a testable framework, but it does not provide a formal meta-analysis or certainty-of-evidence grade. Conclusions are therefore framed according to directness of measurement and biological plausibility rather than pooled effect estimates.

## 10 Conclusion

Insulin resistance cannot be localized from plasma insulin or intracellular signaling alone. Before insulin activates a target cell, it must be delivered through the microvasculature, cross the endothelium, distribute through tissue, and persist despite organ-specific clearance. Diabetes and obesity can disturb several of these processes at once, producing temporal and tissue-specific mismatches between circulating insulin and effective tissue exposure.

The strongest evidence supports a coupled model rather than a single universal interstitial lesion. Vascular delivery and insulin clearance are established components of insulin physiology; endothelial transport now has increasingly strong mechanistic support from IR/IRS and trafficking studies; and interstitial diffusion remains plausible but comparatively under-measured. The immediate value of the insulin-access continuum is experimental. It makes otherwise hidden intermediate steps explicit, reduces the risk of mistaking a surrogate change for proof of mechanism, and defines measurements that can falsify the hypothesis. The next decisive advance will come from synchronized, tissue-specific studies that quantify delivery, interstitial exposure, clearance, cellular signaling, and metabolic response in the same participants.

## Data Availability

The original contributions presented in the study are included in the article/supplementary material. Further inquiries can be directed to the corresponding author.
